# How is health equity considered in policy evaluations employing quasi-experimental methods? A scoping review and content analysis

**DOI:** 10.1093/eurpub/ckae188

**Published:** 2024-11-27

**Authors:** Kerstin Sell, Setareh Rabbani, Jacob Burns

**Affiliations:** Chair of Public Health and Health Services Research, IBE, Faculty of Medicine, LMU Munich, Munich, Germany; Pettenkofer School of Public Health, Munich, Germany; Chair of Public Health and Health Services Research, IBE, Faculty of Medicine, LMU Munich, Munich, Germany; Pettenkofer School of Public Health, Munich, Germany; Professorship of Public Health and Prevention, TUM School of Medicine and Health, Technical University of Munich, Munich, Germany; Professorship of Public Health and Prevention, TUM School of Medicine and Health, Technical University of Munich, Munich, Germany

## Abstract

Public health researchers employ quasi-experimental methods (QEM) to evaluate the effects of policies. Whilst some policies are designed to improve (health) equity, others may intentionally or unintentionally have detrimental effects on disadvantaged populations. We thus sought to investigate how health equity is addressed in policy evaluations which employ QEM. We conducted a content analysis on studies sourced from a scoping review. We drew a random sample of 350 records identified in systematic database searches in Medline, EMBASE, and EconLit (December 2022). Studies that employed QEM labels and examined public policies implemented in the WHO European region were included. We extracted data on study design, policies, and populations; assessed whether outcomes were examined in population sub-groups (as defined by PROGRESS-Plus criteria); and analysed discussion sections for equity-related conclusions. We included 59 studies, of which 39 (66.1%) studies considered health equity—albeit to variable depth. Twenty-five studies were focused exclusively on examining policy outcomes in a disadvantaged population (42.4%), of which 19 studies evaluated policies that targeted disadvantaged groups (e.g. minimum wage, social housing policies). Outcomes were stratified for one or more sub-populations in 22 studies (37.3%), most commonly for gender (*n* = 15, 25.4%) and a measure of socio-economic status (*n* = 13, 22%), particularly income and employment. Equity-related results and implications were discussed in 24 studies. While policy evaluations employing QEM have considerable value for informing decision-making in public health and other sectors that influence health, their potential to investigate equity impacts is currently not harnessed.

## Introduction 

The health of populations is strongly influenced by the environments in which people grow up, live and work and the social determinants of health [1, 2]. Consequently, policies implemented by many sectors—for example, the housing, food production, and transport sectors—have an impact on population health. Policy-based interventions targeting these ‘upstream’ determinants of health are more effective at improving health and reducing health inequities than ‘downstream’ interventions that require substantial individual agency [3, 4]. Aiming to support evidence-informed policymaking, public health researchers hence take a strong interest in employing and advancing rigorous policy evaluation methods [5, 6].

Increasingly, they employ quasi-experimental methods (QEM) to evaluate the effects of policies [7]. This set of methods originates from econometrics and helps researchers to evaluate the causal effect of an intervention, where it is not possible, feasible, or appropriate to conduct a randomized controlled trial (RCT), as is typically the case for policies [8, 9]. The term QEM is, however, conceived differently in different disciplines and research traditions [10]. Given the variable use of these labels, the studies may substantially vary in methodological strength. Lately, others have therefore recommended foregoing these labels and using ‘as if randomization’ as a criterion of study strength and conceptualizing these policy evaluations as ‘natural experiments’ [9, 11, 12].

Another methodological concern is appropriate approaches to evaluate policy effects on health equity (HE) [13]. According to the World Health Organization (WHO), health inequity is defined as ‘systematic differences in the health status of different population groups’ [14] which are unfair, avoidable, or remediable [15]. Health inequity arises due to inequalities in socio-economic factors [16] and exists between and within countries [2]. Health inequalities, in contrast, are understood as differences in health outcomes that are not deemed ‘unfair’ [17]. (This terminology is used by WHO; however, in parts of Europe, particularly the UK, ‘health inequality’ and ‘health inequity’ are used interchangeably [18].) Action on (health) inequity is grounded in human rights principles; central to WHO’s efforts [14, 19, 20]; and understood to contribute to improved overall population wellbeing [21].

Policy evaluation can delineate where policies contribute to maintaining or widening (health) inequities, or when policies have unintended negative effects on disadvantaged groups [22].

Thus, there have been widespread calls to improve consideration of HE in epidemiological studies, particularly those directly intended to inform decision-making [19, 23, 24]. To date, however, (health) equity is under-considered in much of the research focusing on intervention effects, including in RCTs [25] and systematic reviews [16]. In policy evaluation, while multiple analytical methods exist to examine HE [8] this research still appears to be in its ‘infancy’ [13].

In a methodological study examining policy evaluations employing QEM [26] questions regarding consideration of HE in this particular set of methods emerged.

### Objectives

We sought to (1) identify and characterize a sample of policy evaluation studies employing QEM to evaluate health and social policies implemented in the WHO European region and (2) to examine how HE is considered in these studies.

## Methods

We undertook a scoping review and subsequent content analysis of a random sample of identified articles. The scoping review was part of a larger project examining methodological characteristics of QEM employed in policy evaluation [26]. While we do not report the methodological work here, we report basic characteristics of the sample identified in the scoping review and hence draw on the PRISMA Extension for Scoping Reviews to report our methods and results [27].

### Design and conceptual framework

We drew on the PROGRESS-Plus framework to conceptualize HE. The framework supports researchers in identifying socially stratifying factors (SSFs) which shape opportunities and health outcomes, i.e. place of residence, race/ethnicity/culture/language, occupation, gender/sex, religion, education, socio-economic status (SES), and social capital; which make up the acronym ‘PROGRESS’, and further characteristics associated with discrimination (‘Plus’) [28, 29]. SSFs stratify populations into more or less advantaged groups, which may vary by context (e.g. which religion is associated with greater advantage).

To investigate how HE was considered, we examined (i) whether *policies* focused on disadvantaged populations, (ii) whether *studies* focused exclusively on disadvantaged populations (e.g. the effects of premature labour policy investigated in low income mothers only (see [72], Supplementary Material S2).), (iii) whether studies examined outcomes across sub-populations (stratification); and (iv) whether authors discussed HE-related aspects.

When identifying policies or studies which focused on disadvantaged populations (i and ii), we considered young or old age and disability as additional criteria of potentially disadvantaged groups (‘PROGRESS-Plus’) [29, 30]. We did not consider age as equity-relevant stratification when outcomes were merely disaggregated by age categories.

### Data sources, sampling, and eligibility

In the scoping review, we searched Medline, EMBASE, and EconLit databases in December 2022. Our search strategies were published alongside our protocol [26]. After deduplication, we drew a random sample of 350 articles, which we screened for eligibility in Rayyan [31]. We included peer-reviewed articles published after 2010 that evaluated policies implemented in the European region, as defined by WHO, employing QEM, i.e. studies labelled as regression discontinuity (RD), (controlled) interrupted time series (cITS/ITS), synthetic control (SC), difference-in-differences (DiD), and controlled before-and-after studies (CBA) [26].

### Data charting and analysis

We developed a Microsoft Excel-based data extraction sheet, which was trialled and refined by two researchers (K.S., S.R.). Data extraction categories included basic study characteristics, study design features, detail on policies, and populations. For some categories, data extraction entailed simple document searches for keywords (e.g. equit*, equal*); for other categories it entailed straightforward data extraction (e.g. of the policy title, year of policy implementation); and for other categories, it entailed some interpretation (e.g. policy area) or analysis (e.g. on stratification). To examine how HE was discussed (iv), we extracted larger segments of text referring to differences in health outcomes in sub-groups and other equity-related aspects from the Discussion and Conclusion sections.

We analysed categorical data (e.g. country, QEM label) descriptively and summarized our findings in narrative and tabular format.

We analysed narrative data using abridged procedures for qualitative content analysis as described by Schreier [32]. This involved development of a preliminary codebook. After familiarization with the data, one author (S.R.) marked quotations, and applied *a priori* codes deductively. Further codes and higher-level categories were developed inductively. After a subset of all data was coded, a second author reviewed the coding (K.S.), leading to subsequent adaptation of the codebook. Subsequently, one author (S.R.) applied the codebook to the entirety of included material. Results were summarized narratively, by category.

To enhance trustworthiness of our findings, the full data extraction table was reviewed by a second researcher (K.S.) and qualitative content analysis included iterative rounds of review and discussion between authors.

### Ethics

Since all included data were publicly available, we did not seek approval from an ethics committee.

## Results

### Study sample

**Figure 1.**
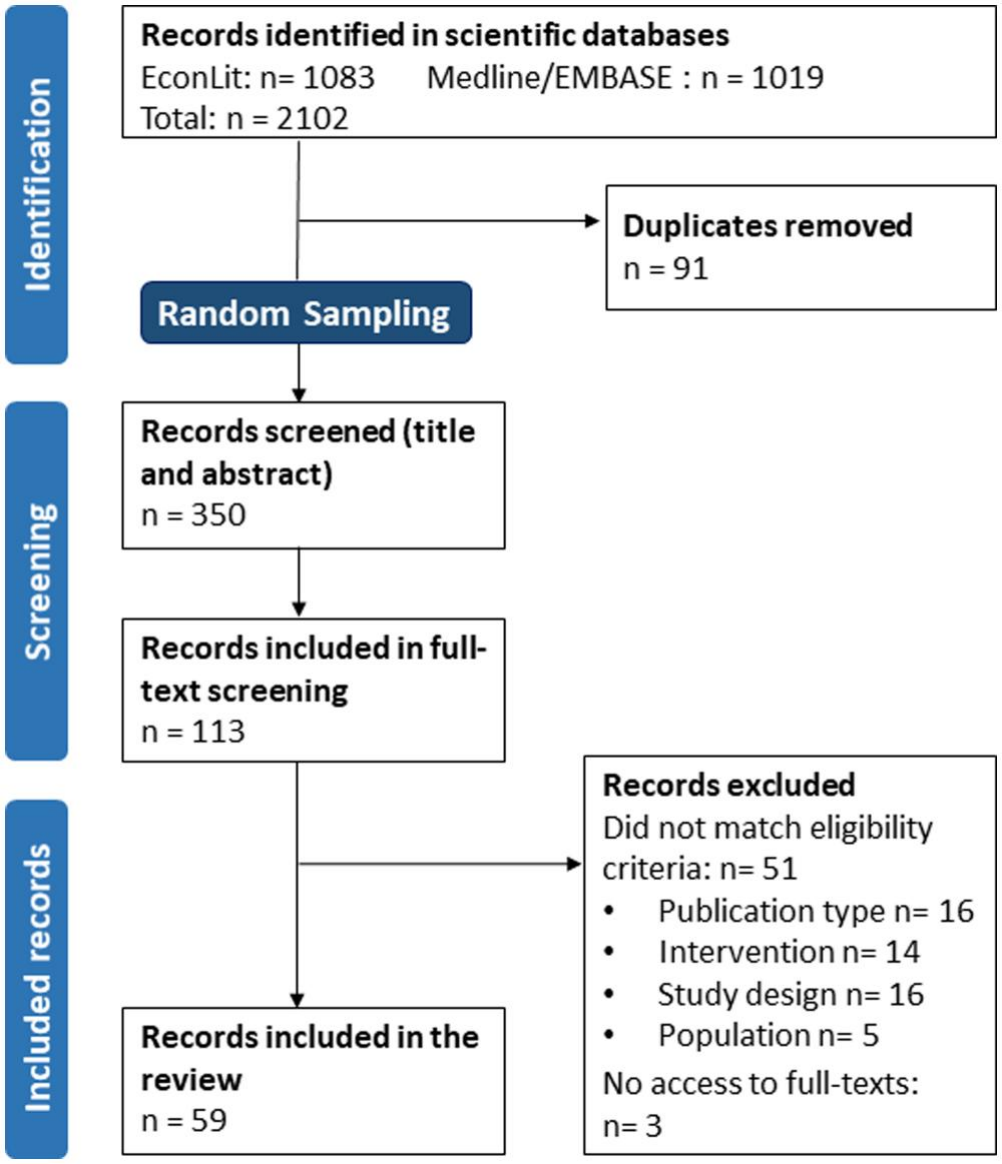
Identification of study sample.

Database searches rendered 2102 results and our final study sample consisted of 59 studies (Fig. 1). One study has been retracted since we conducted our search [33]. We included the revised publication instead [34].

Policies implemented in 23 European countries were evaluated (Table 1, continued reference list in Supplementary Material S1). Most studies evaluated policies implemented in the UK (*n* = 16) [34–49] and European Union (EU, *n* = 33), e.g. Germany [50–54], France [55–58], Spain [59–62], Ireland [63–65], Italy [66–68], Sweden [69–71], and the Netherlands [72–74]. Countries outside of the EU included Georgia [75, 76], Russia [77, 78], Switzerland [79–81], Israel [82], Norway [83], and Ukraine [84]. Three studies examined the effect of the policies implemented at the supranational level or in more than one country [80, 85, 86].

**Table 1.** Summary of included studies

First author, year of publication	Discipline (journal)	QEM label^b^	Country	Policy description	Population targeted by policy	Population in which outcomes were assessed (if different)
Ahlfeldt 2018 [50]	Economics	DiD	Germany	Minimum wage policy	Low-wage workers/employers	Labour market participants
Akbulut 2017 [51]	Economics	DiD	Germany	Mandatory employment for post-war reconstruction	‘Rubble women’ (women born between 1920 and 1934)	–
Anderson 2022 [39]	Public health	ITS	UK^a^	Lockdown	General population	–
Angelini 2019 [35]	Public health	RD	UK	Winter fuel payment	Households with one person over 60	–
Anger 2011 [52]	Health economics	DiD	Germany	Indoor smoking ban	General population	–
Armeni 2016 [66]	Health economics	DiD	Italy	Co-payments, prescription quotas, and therapeutic reference pricing	General population	–
Avram 2018 [40]	Economics	DiD	UK^a^	Reform of unconditional income support for single parents	Single parents	–
Bargain 2012 [63]	Economics	DiD	Ireland	Divorce legalization	Married couples	–
Ben Lakhdar 2016 [56]	Health economics	DiD	France	Cigarette tax increase	General population	–
Biro 2019 [88]	Public health	DiD	Hungary	Incentives for using ICD codes in antibiotic prescribing for children	Physicians	Children aged 0–4
Boes 2015 [79]	Health economics	DiD	Switzerland	Indoor smoking ban	General population	–
Braakmann 2014 [41]	Other	DiD	UK^a^	Depenalization of cannabis possession and consumption	General population	General population (10–25 years)
Bratberg 2020 [83]	Health economics	DiD	Norway	Reduced workload for older teachers	Teachers in public schools	–
Cecil 2015 [42]	Public health	CBA	England	Primary care policy (physicians opting out of responsibility for out-of-hours care)	Physicians	Children under 15 years
Chyderiotis 2019 [55]	Public health	CBA	France	Smoking ban in public places	Smokers	–
Clark 2022 [57]	Economics	DiD	France	Increase in layoff tax for older workers	Employees in private sector	–
Daysal 2019 [72]	Health economics	RD	Netherlands	Perinatal medical care for premature labour	Pregnant women, medical professionals	Low-income, low-risk pregnant women
De Jorge-Huertas 2021 [59]	Economics	ITS	Spain	Homeownership laws, e.g. land regime changes	General population	–
Dearden 2014 [43]	Economics	DiD	UK^a^	Student aid (maintenance grants)	University students from low-income families	–
Dumeignil 2022 [80]	Other	DiD	EU and Switzerland	Agreement on the free movement of persons	European cross-border workers	General population
Fiorio 2010 [67]	Economics	DiD	Italy	Changing co-payment levels	General population	–
Focacci 2020 [68]	Economics	DiD	Italy	Active labour market policy, e.g. internships, on-the-job training, apprenticeships, support for self-employment, international mobility	Young adults under 30	–
Gambaryan 2018 [77]	Public health	SC	Russia	Indoor and outdoor smoking ban, progressive tobacco tax, advertising bans, warning on packaging, information campaigns	Smokers	General population
García-Pérez 2019 [60]	Economics	RD and DiD	Spain	Liberalization of fixed-term employment contracts	Labour market participants	Low-skilled adolescents
Gaughan 2019 [44]	Health economics	DiD and SC	England	Same-day discharge bonus policy	Healthcare providers	General population
Gibbons 2020 [36]	Economics	DiD	UK	Under-occupancy penalty (‘bedroom tax’)	Social housing tenants	–
Grabovac 2018 [91]	Public health	SC	Austria	Regulation of trans fatty acids	General population	–
Grenet 2013 [85]	Economics	RD	UK^a^, France	Raising of the minimum school-leaving age	Students (14–16)	General population (aged 25–60)
Haghpanahan 2019 [45]	Public health	DiD	Scotland	Decrease in blood alcohol concentration limits for drivers	General population	–
Hamilton 2014 [92]	Public health	ITS	UK	(Re-)classification of cannabis in criminal law	Individuals indicted for cannabis use	Hospital patients admitted for cannabis psychosis
Hengel 2021 [73]	Health economics	RD	Netherlands	Penalized early retirement	General population (retirement age)	–
Honkaniemi 2022 [69]	Public health	ITS	Sweden	Incentives for fathers’ leave uptake	Fathers	–
Kaliskova 2014 [90]	Economics	DiD	Czech Republic	Joint taxation	Married couples	–
Kleif 2020 [89]	Public health	RD	Denmark	Compulsory educational programme for unemployed young adults	Low-skilled unemployed young adults	–
Kümpel 2019 [53]	Health economics	DiD and SC	Germany	Extended reimbursement for nursing homes during resident’s absence	Nursing home providers	Hospital patients discharged to nursing homes
Lavikainen 2020 [87]	Public health	ITS	Finland	Decrease in reimbursement level of non-insulin antidiabetic medications	Type 2 diabetic patients	–
Maynou 2019 [61]	Health economics	ITS	Spain	Introduction of 'euro per prescription' co-payment	General population	–
McDonnell 2022 [64]	Public health	DiD	Ireland	Free GP visit for children under 6 (phased UHC roll-out)	Parents, children under 6, healthcare providers	Children under 6
Mohan 2017 [46]	Public health	DiD and CBA	Northern Ireland	Neighbourhood renewal	General population (aged 16 and over)	–
Muravyev 2016 [84]	Economics	DiD	Ukraine	Mandatory Ukrainian language school exit test	Minority language high school students (final year)	Final year high school students
Nedberg 2022 [76]	Health economics	ITS	Georgia	Penalty for missing caesarean section rate reduction targets	Healthcare providers	Pregnant women
Pettersson 2012 [70]	Public health	ITS	Sweden	New reimbursement scheme for glucose-lowering therapy	Patients with prescriptions for glucose-lowering therapy	General population
Popham 2015 [37]	Public health	DiD	UK	‘Right to Buy’ policy	Social housing tenants (households eligible for policy)	–
Reeves 2017 [38]	Health economics	DiD	UK	Minimum wage legislation	Low-wage workers	–
Reinhold 2013 [86]	Economics	DiD	Multiple	Legalization of unilateral divorce	General population (adults)	Individuals growing up under unilateral divorce laws
Rogers 2023 [34]	Public health		UK	Soft drinks industry levy	General population	–
Runst 2020 [71]	Economics	DiD and SC	Sweden	Carbon taxation	General population	–
Saffer 2012 [58]	Economics	DiD	France	Decreased working hours (from 39 to 35 h for a full-time work week without any reduction in salary)	General population (in employment)	–
Serrano-Alarcon 2022 [47]	Health economics	DiD	England	Lifting of lockdown policy (Stay at Home)	General population	–
Shelkova 2020 [78]	Economics	DiD	Russia	Non-cash subsidy for mothers having a second or higher order child (e.g. for improvement of the family’s living conditions)	Mothers/couples	Men
So 2021 [48]	Public health	DiD	Scotland	Minimum unit pricing	General population (adults)	Hospital and ED patients
Stallings-Smith 2013 [65]	Public health	ITS	Ireland	Workplace and indoor smoking ban	General population	General population (adults over 35)
Szatkowski 2011 [49]	Other	ITS	England	Workplace and indoor smoking ban	General population	–
Temkin 2022 [82]	Public health	ITS	Israel	Face mask regulations	Healthcare workers and visitors	Health care workers
Troelstra 2016 [74]	Public health	ITS	Netherlands	Multiple tobacco control policies	General population	–
Vall Castello 2012 [62]	Economics	DiD	Spain	Tax deduction for disabled women	Disabled women/employers	Disabled women
Wicki 2011 [81]	Other	ITS	Switzerland	Prohibition of off-premise alcohol sales (e.g. takeaways, supermarkets, or kiosks) at night; and prohibition of sales at gas stations	General population	–
Xu 2022 [54]	Health economics	DiD	Germany	Abolition of co-payment for ambulatory care	General population	General population (over 50)
Zoidze 2013 [75]	Public health	DiD	Georgia	Public–private partnership for health insurance for poor households	Low-income households	–

QEM = quasi-experimental method, DiD = difference-in-differences study, ITS/cITS = (controlled) interrupted time series study, RD = regression discontinuity study, SC = synthetic control study, CBA = controlled before-after study, ICD = International Classification of Diseases, UHC = universal health coverage.

a Two or three UK countries, e.g. England and Wales.

b QEM label as employed by study authors.

Just over half of the studies (*n* = 30) examined health policies, including nine studies related to healthcare financing [53, 54, 61, 66, 67, 70, 75, 87, 88], 11 on tobacco or alcohol control [45, 48, 49, 52, 55, 56, 65, 74, 77, 79, 81], and five focusing on healthcare services [42, 44, 64, 72, 76].

An additional 26 studies examined ‘social protection and welfare’ policies, i.e. including studies assessing policies on employment (*n* = 11) [38, 50, 51, 57, 58, 60, 62, 68, 73, 83, 89], family life (*n* = 6) [40, 63, 69, 78, 86, 90], housing (*n* = 4) [35–37, 59], and education (*n* = 3) [43, 84, 85].

Studies were published in public health journals (*n* = 22, 37%), health economics (*n* = 14, 24%) and economics journals (*n* = 19, 32%; Table 1).

Three studies (5%) cited a published or registered protocol [33, 48, 69] and five studies (8%) were part of a larger policy evaluation [46, 48, 55, 69, 74]. Only one study (1.7%) provided a logic model [35].

### Equity consideration in included studies

Overall, (health) equity aspects were considered in 39 studies (66.1%; Table 2). This included 19 studies (32.2%) in which the policy was focused on a disadvantaged population per se and an additional six studies in which policy outcomes were investigated exclusively in a disadvantaged population (*n* = 25, 42.4%). Stratification was used in 22 studies (37.3%).

**Table 2.** Consideration of (health) equity aspects in study scope, analysis, discussion, and recommendations

First author, year of publication	Policy category and sub-category	Study HE aim?	Policy focused on disadvantaged populations?	Study focused on disadvantaged populations?	Study outcomes stratified for socially stratifying factors?				HE disc.?	HE recommendations?	Equity considered?
					Sex/gender	Race/ethnicity	Place of residence	Any SES			
Ahlfeldt 2018 [50]	Social policy (SP): Employment	No	Yes	Yes	No	No	No	No	No	No	Yes
Akbulut 2017 [51]	SP: Employment	No	Yes	Yes	NA	No	No	No	No	No	Yes
Anderson 2022 [39]	Health policy (HP): COVID-19 regulation	No	No	No	No	No	No	Yes	Yes	Yes	Yes
Angelini 2019 [35]	SP: Housing	No	Yes	Yes	No	No	No	Yes	Yes	Yes	Yes
Anger 2011 [52]	HP: Tobacco control	No	No	No	No	No	No	No	No	No	No
Armeni 2016 [66]	HP: Healthcare financing	No	No	No	No	No	No	No	No	No	No
Avram 2018 [40]	SP: Family	Yes	Yes	Yes	No	No	No	No	No	No	Yes
Bargain 2012 [63]	SP: Family	No	Yes	Yes	NA	No	No	No	No	No	Yes
Ben Lakhdar 2016 [56]	HP: Tobacco control	No	No	No	No	No	No	No	No	No	No
Biro 2019 [88]	HP: Healthcare financing	Yes	Yes	Yes	No	No	Yes	Yes	Yes	Yes	Yes
Boes 2015 [79]	HP: Tobacco control	No	No	No	No	No	No	No	No	No	No
Braakmann 2014 [41]	crime and safety	No	No	No	No	No	No	No	No	No	No
Bratberg 2020 [83]	SP: Employment	No	No	No	Yes	No	No	No	Yes	No	Yes
Cecil 2015 [42]	HP: Healthcare service	Yes	No	Yes	No	No	No	No	Yes	No	Yes
Chyderiotis 2019 [55]	HP: Tobacco control	No	No	No	No	No	No	No	No	No	No
Clark 2022 [57]	SP: Employment	Yes	Yes	Yes	Yes	No	No	Yes	No	No	Yes
Daysal 2019 [72]	HP: Healthcare service	Yes	No	Yes	No	No	No	No	No	Yes	Yes
De Jorge-Huertas 2021 [59]	SP: Housing	No	No	No	No	No	No	No	No	No	No
Dearden 2014 [43]	SP: Education	Yes	Yes	Yes	No	No	No	NA	Yes	Yes	Yes
Dumeignil 2022 [80]	SP: Migration	No	No	No	NA	NA	NA	No	No	No	No
Fiorio 2010 [67]	HP: Healthcare financing	No	No	No	No	No	No	No	No	No	No
Focacci 2020 [68]	SP: Employment	Yes	Yes	Yes	Yes	No	No	No	Yes	Yes	Yes
Gambaryan 2018 [77]	HP: Tobacco control	No	No	No	No	No	No	No	No	No	No
García-Pérez 2019 [60]	SP: Employment	Yes	No	Yes	No	No	No	No	Yes	No	Yes
Gaughan 2019 [44]	HP: Healthcare service	No	No	No	No	No	No	No	No	No	No
Gibbons 2020 [36]	SP: Housing	Yes	Yes	Yes	No	No	No	No	Yes	No	Yes
Grabovac 2018 [91]	HP: Food	No	No	No	Yes	No	No	No	Yes	No	Yes
Grenet 2013 [85]	SP: Education	No	No	No	Yes	No	No	No	No	No	Yes
Haghpanahan 2019 [45]	HP: Alcohol control	No	No	No	No	No	No	No	No	No	No
Hamilton 2014 [92]	Crime and safety	No	No	No	No	No	No	No	No	No	No
Hengel 2021 [73]	SP: Employment	Yes	No	No	Yes	No	No	Yes	Yes	Yes	Yes
Honkaniemi 2022 [69]	SP: Family	Yes	No	Yes	NA	Yes	No	No	Yes	Yes	Yes
Kaliskova 2014 [90]	SP: Family	No	No	No	Yes	No	No	No	Yes	Yes	Yes
Kleif 2020 [89]	SP: Employment	No	Yes	Yes	No	No	No	No	No	No	Yes
Kümpel 2019 [53]	HP: Healthcare financing	No	Yes	Yes	No	No	No	No	No	No	Yes
Lavikainen 2020 [87]	HP: Healthcare financing	No	No	No	No	No	No	No	No	No	No
Maynou 2019 [61]	HP: Healthcare financing	No	No	No	Yes	No	No	Yes	Yes	No	Yes
McDonnell 2022 [64]	HP: Healthcare service	No	Yes	Yes	No	No	No	No	No	No	Yes
Mohan 2017 [46]	SP: Health promotion	No	Yes	Yes	Yes	No	No	Yes	Yes	Yes	Yes
Muravyev 2016 [84]	SP: Education	Yes	No	Yes	No	No	Yes	Yes	No	No	Yes
Nedberg 2022 [76]	HP: Healthcare service	No	Yes	Yes	NA	No	No	No	No	No	Yes
Pettersson 2012 [70]	HP: Healthcare financing	No	No	No	No	No	No	No	No	No	No
Popham 2015 [37]	SP: Housing	No	Yes	Yes	No	No	No	No	No	No	Yes
Reeves 2017 [38]	SP: Employment	Yes	Yes	Yes	No	No	No	No	Yes	No	Yes
Reinhold 2013 [86]	SP: Family	No	No	No	No	No	No	Yes	No	No	Yes
Rogers 2023 [34]	HP: Food	No	No	No	No	No	No	No	Yes	No	No
Runst 2020 [71]	Environment	No	No	No	No	No	No	No	No	No	No
Saffer 2012 [58]	SP: Employment	No	No	No	Yes	No	No	No	No	No	Yes
Serrano-Alarcon 2022 [47]	HP: COVID-19 regulation	No	No	No	Yes	No	No	Yes	Yes	Yes	Yes
Shelkova 2020 [78]	SP: Family	Yes	No	No	No	No	No	No	Yes	Yes	Yes
So 2021 [48]	HP: Alcohol control	Yes	No	No	Yes	Yes	No	Yes	Yes	Yes	Yes
Stallings-Smith 2013 [65]	HP: Tobacco control	No	No	No	Yes	No	No	No	No	No	Yes
Szatkowski 2011 [49]	HP: Tobacco control	No	No	No	Yes	No	No	Yes	Yes	Yes	Yes
Temkin 2022 [82]	HP: COVID-19 regulation	No	No	No	No	No	No	No	No	No	No
Troelstra 2016 [74]	HP: Tobacco control	No	No	No	No	No	No	No	No	No	No
Vall Castello 2012 [62]	SP: Employment	Yes	Yes	Yes	Yes	No	No	No	Yes	Yes	Yes
Wicki 2011 [81]	HP: Alcohol control	No	No	No	No	No	No	No	No	Yes	No
Xu 2022 [54]	HP: Healthcare financing	Yes	No	Yes	No	No	No	Yes	Yes	Yes	Yes
Zoidze 2013 [75]	HP: Healthcare financing	Yes	Yes	Yes	No	No	No	No	Yes	Yes	Yes
*n*		18	19	25	15	2	2	13	24	18	39

In 18 studies (30.5%), authors explicitly mentioned a (health) equity-related aim, e.g. by making explicit which sub-populations they considered:

This study investigated the effects of a national early retirement reform, which […] penalized early retirement, on paid employment and different exit pathways and examined whether these effects differ by gender, income level and health status. [73]

#### Policies targeting disadvantaged populations

Of the 19 studies examining policies primarily focused on disadvantaged populations, some investigated direct policy effects on these groups, e.g. effects of minimum wage policy for low-wage workers [50] or winter fuel payment for the elderly [35]. Others investigated potential unintended negative effects, e.g. the effects of the under-occupancy penalty in social housing (‘bedroom tax’) [36]. Some authors studied complex effects, e.g. increased layoff taxes which benefit older workers but affect younger workers negatively [57].

#### Study population: focus on disadvantaged groups

In the 25 studies exclusively focused on disadvantaged groups, authors studied effects in women [51, 63, 76], the elderly [35, 53, 54], children [42, 64, 86, 88], ethnic and language minorities [69, 84], and populations disadvantaged by SES, i.e. low-wage, low-skilled, or younger workers [38, 50, 57, 60], social housing tenants [36, 37], and deprived neighbourhoods [46].

Some studies had an intersectional focus, investigating policy effects in groups disadvantaged by two or more SSFs. This included disabled women [62], low-income mothers [72], and unemployed youth [68, 89].

#### Study outcomes: stratification

Outcomes in different sub-populations were examined in 22 studies (37.3%) by stratifying for one or more SSFs. Outcomes were most commonly stratified for gender (*n* = 15, 25.4%) and a measure of SES (*n* = 13, 22%), in particular income and employment. In two studies, respectively, outcomes were stratified for place of residence and ethnicity (Tables 2 and 3). We did not identify any studies stratifying for occupation (other than employment status), religion, or social capital.

**Table 3.** Stratification for PROGRESS-Plus criteria

	Stratified (*n*)	%
Any	22	37.3
Place of residence^a^	2	3.4
Race/ethnicity/culture/language	2	3.4
Occupation	0	0
Gender/sex	15	25.4
Religion	0	0
Education	3	5.1
SES (any)^b^	13	22
Income	7	11.9
Employment^c^	5	8.5
Social class	1	1.7
Other SES^d^	7	11.9
Social capital	0	0

a Urban/rural.

b Cumulative score including the subsequent categories and education.

c For example, employment status, (un)employment rate, husband’s employment, retirement.

d For example, area deprivation, housing tenure/standard, subjective financial status, GDP per capita.

Outcomes were also commonly stratified for age (*n* = 13, 22.0%, not included in Table 3). In four studies, age constituted the only stratifying factor. No study stratified for ability.

#### Study conclusions

Of the 39 studies that considered equity aspects, 24 discussed these (61.5%; Table 2). Explicit discussion of equity implications was rare in the studies which exclusively focused on a disadvantaged population but did not stratify outcomes for further sub-groups. Of the 22 studies stratifying for PROGRESS-Plus criteria, these results were discussed in 15 studies (68.2%). Outcomes stratified by SES were most commonly discussed (*n* = 10, 76.9% of studies that stratified for SES); stratification by sex/gender less commonly (*n* = 5, 33.3% of studies that stratified for sex).

In a study that examined alcohol purchases during the COVID-19 lockdown in Great Britain, stratification helped to delineate diverging trends in sub-populations, while there was no change in overall purchasing behaviour (compared to previous years) in the aggregated data:

There was some evidence to suggest that the most disadvantaged households increased their purchases more than the least disadvantaged households, based on social grade and deprivation index, and, to some extent, on household income. [39]

Authors of 10 studies drew explicit conclusions about the (health) equity impact of policies with reference to ‘equity’ (*n* = 1) and ‘(in)equality’ (*n* = 9):

This development may indicate an adverse impact of widened inequalities as a result of unintended consequences for children and other groups. [42]

In other studies, authors eschewed these terms but still clearly named inequitable policy effects on disadvantaged populations:

The poorest and most vulnerable are most at risk of harm from alcohol consumption and tend to consume such cheap alcohol; therefore, MUP (minimum unit price) would be of greater benefit to them than other drinkers. [48]

Eighteen studies provided equity-related recommendations for policymakers. These included calls to policymakers to focus more on disadvantaged groups, e.g. through improved evaluation of differential policy effects [35, 54, 72, 73], and more attention to unintended or systems effects of policies [54, 78, 90].

[…] the current study offers a word of caution for policy-makers. They need to be aware of the societal and individual side effects of these kind of reforms across different groups when implementing reforms prolonging working lives. [73]

Researchers further recommended better implementation (e.g. enforcement, outreach) [62, 68, 88], policy improvement (e.g. focussing of resources on disadvantaged groups) [46, 49, 75], and suggested complementary policies to protect or improve health [39, 46, 47, 62, 75].

## Discussion

### Principal findings

In our scoping review, we identified a large number of studies using QEM to evaluate policies implemented in the WHO European Region. In 39 of the 59 included studies, one or more equity aspects were considered. Equity was considered in variable depth; in 19 studies, authors examined policies targeting disadvantaged populations and in an additional six studies they focused exclusively on the effects of untargeted policies in disadvantaged populations. In 22 studies, outcomes were stratified for one or more SSFs, most commonly gender or an SES measure. Equity-related findings were discussed in 24 articles which fed into recommendations for policymakers in 18 articles, including calls to improve monitoring and evaluation of policy effects and better conceptualizing of unintended effects. Authors of 10 studies explicitly mentioned equity or equality. Very few studies drew on a published or registered study protocol or used a logic model.

### Results in the context of other literature

Given the longstanding calls for better consideration of equity in research aimed to inform policymaking [24], there exist a few studies with a scope similar to ours [13, 16, 23–25, 93]. In contrast to some of this research, our sample was defined by the methodological label, not an equity focus of included studies. Others examined studies which specifically assessed HE or were defined as equity-relevant [13, 23–25]. Our conceptualization of the research corresponded with their approaches, charting whether studies focused on disadvantaged populations, determining which sub-group analyses were undertaken, and whether equity-related conclusions were drawn; e.g. in systematic reviews of effectiveness [16], and (cluster-)RCTs [25]. In one methodological review, a substantial list of further equity aspects was examined in an effort to provide a baseline for reporting of equity in observational studies [23].

Across these studies, authors identified rates of sub-group analysis for PROGRESS-Plus criteria that were similar or lower than the rates we identified. In ‘equity-relevant’ [94] (cluster-)RCTs, 37% of included studies provided sub-group analysis for at least one PROGRESS-Plus criterion [25]. In systematic reviews, 26 out of 158 reviews (16.5%) included sub-group analysis [16]. Age, gender/sex, race/ethnicity, and SES were the SSFs most commonly considered [16, 23–25]. Only in one review, place of residence constituted the most commonly assessed SSF [13]. The former corresponds with our results, except race/ethnicity being considered less commonly in our sample, which may be explained by our focus on Europe (excluding studies from the USA in which stratification by race/ethnicity is more common [23]). One study specifically examined analytical approaches to evaluate policy effects on HE and identified mostly observational studies, many of which included statements about causal attribution despite their mostly cross-sectional design [13].

It is worth noting that despite a proportion of studies examining outcomes in equity-relevant sub-groups, the majority of studies included in our review and in similar research—some specifically including equity-relevant research—did not examine outcomes in disadvantaged groups. This is worrying, given that the social and gender gradient in health outcomes is well established in public health [1, 2, 20, 95]. In our study, inclusion of Economics studies (in which social determinants may be a less established concept) may partly explain this.

Other explanations for this under-consideration of equity are technical, statistical, and conceptual.

Technical obstacles relate to lack of access to or availability of sub-population data. While this is a common challenge in quasi-experimental research, which commonly draws on secondary data sources, this information was available in a large number of studies in our sample [baseline differences between sub-populations reported in 42 studies (71.2%)]. This was also observed in the methodological study on equity-relevant (cluster-)RCTs: ‘We found that even when the data are available, opportunities to analyse HE considerations are frequently missed’ [25].

While technical obstacles relate to both studies focused on disadvantaged populations and those using stratification, statistical challenges relate particularly to studies employing stratification. Sub-group analysis may be hindered due to issues relating to sample size, statistical power, and multiple testing. These require careful consideration, and, importantly, pre-specification in a protocol [96]. In an effort to balance tensions between statistical challenges and policymakers’ need for evidence on ‘what works to close the gap in health between socio-economic groups’, Hu *et al.* provide in-depth guidance to help researchers choose from a set of analytical methods which enable evaluation of policy effects on health inequalities, either by stratifying outcomes or using an interaction term [8].

Conceptual challenges hindering better consideration of equity in policy evaluation likely relate to envisioning the potentially complex equity effects of policies. Equity implications of policies may vary, depending on the measure. Exposure to health risks and unhealthy environments are stratified across socio-economic groups. Policies targeting these may thus affect all groups equally, affect advantaged groups more positively (leading to increasing inequity), or affect disadvantaged groups more positively (leading to decreasing inequity). For certain policy measures, these effects are well established but others require careful conceptualization of the pathways of how the policy may affect health and/or social determinants, including unintended effects [22]. Frameworks such as PROGRESS-Plus [28, 29], numerous WHO resources [19], and tools for specific policy areas, such as obesity prevention [97], have been developed to support consideration of equity in policy development and evaluation. It has been noted that a tool like PROGRESS-Plus will help users to consider HE in an evaluation [28] but that it ‘does not *ensure* critical thinking’ (emphasis added) [30]. One way forward to improve critical thinking and conceptualization of complex equity impacts is the use of logic models, which include ‘theoretically plausible mechanisms for a reduction on inequalities in health’ [8]. We noted, however, that only one study included in our review incorporated a logic model [35], which was also observed in other research [13]. Thus, more theoretically grounded approaches to aid the conceptualization of policy effects and equity impacts remain under-utilized.

### Implications for policy, practice, and research

Researchers undertaking policy evaluation should aim to address policymakers’ needs for information on *how* policies affect populations including the least advantaged. Concerns about statistical power when undertaking sub-group analyses are warranted but can be counter-balanced by a number of steps. This includes focusing on the most important populations (by drawing on PROGRESS-Plus or other guidance [8], and development of a logic model); stating planned sub-group analyses in a protocol; and collaborating with statisticians to address sample size challenges.

Researchers further need to explicitly report equity-related results in summary of findings sections, to prevent these findings from ‘disappearing’ in the middle sections of publications. They should include concrete policymaking recommendations, presented in appropriate knowledge translation outputs. Equity-focused research should be explicitly labelled as such so it can be located more easily by research users.

Decision-makers should demand evidence on equity impacts and—ideally—co-develop protocols to provide insights into impacted populations. Importantly, in addition to research employing QEM, decision-making to address health inequities should draw on a plethora of relevant evidence [98].

### Strengths and limitations

We identified a large number of potentially eligible studies and resorted to random sampling to analyse a subset of studies [26], which was done in similar manner in other equity-focused studies [23, 25]. A larger study sample would have made our findings more generalizable but would have prevented us from analysing the included studies at this level of detail. We searched three databases, employing an elaborate search strategy [26]. To ensure high consistency and rigour, we screened all full texts in duplicate and conducted a thorough review of all extracted data. We included studies based on study design *labels* indicating use of QEM. Recognizing the ambiguity of these labels and ongoing methodological debate, it would have been more rigorous to assess whether these labels are accurate based on what authors describe in methods—which was beyond the scope of our research. We examined HE drawing on an established framework [28], supplemented with a qualitative analysis of how equity-related findings were discussed. This analysis goes beyond most similar efforts to assess consideration of (health) equity and provides important insights regarding the studies’ policy relevance. However, we limited our assessment to most, not all PROGRESS-Plus criteria. Newer work informing the equity extension for the STROBE reporting guideline suggests multiple other areas where equity aspects should be considered [23].

## Conclusion

Whilst certain aspects of (health) equity were investigated in studies included in our review, the analysis of equity dimensions remained quite limited in many studies. Implications for policy and practice were not commonly discussed. The potential of policy evaluations employing QEM to inform policymaking and address health inequities is hence not yet harnessed.

## Supplementary Material

ckae188_Supplementary_Data

## Data Availability

The results of our database searches (RIS files) and complete data extraction sheet are available from the first author upon reasonable request. Key points We drew a random sample of records identified in a scoping review to examine how (health) equity is considered in policy evaluations that employ quasi-experimental methods. Given its relevance for political decision-making, policy evaluation should include an equity perspective but this was either absent or limited in most studies. Of 59 included studies, 39 considered at least one aspect of equity but equity-related results were only discussed in 24 studies—of which only 10 explicitly mentioned ‘equity’ or ‘equality’ to describe these. Equity-related recommendations for policy and practice included more attention to unintended policy effects and improved monitoring and evaluation of outcomes in population sub-groups. Only one included study utilized a logic model, a key tool to aid evaluators conceptualize policy effects including equity impacts.
