# Supplementary material for: How is health equity considered in policy evaluations employing quasi-experimental methods? A scoping review and content analysis
Source: Eur J Public Health. 2024 Nov 27;35(1):42–51. doi: 10.1093/eurpub/ckae188 (PMC11832135; doi:10.1093/eurpub/ckae188)
Supplement: ckae188_Supplementary_Data [file ckae188_supplementary_data.zip › ckae188_Supplementary_Data/ejph-2024-08-om-0528-File005.docx]

# Supplement 1: Further references

41. Braakmann N, Jones S. Cannabis depenalisation, drug consumption and crime - Evidence from the 2004 cannabis declassification in the UK. Soc Sci Med. 2014;115((Braakmann, Jones) Newcastle University, Business School-Economics, 5 Barrack Road, Newcastle upon Tyne NE1 4SE, United Kingdom):29–37.

42. Cecil E., Bottle A., Sharland M., Saxena S. Impact of UK primary care policy reforms on short-stay unplanned hospital admissions for children with primary care-sensitive conditions. Ann Fam Med. 2015;13(3):214–20.

43. Dearden L, Fitzsimons E, Wyness G. Money for nothing: estimating the impact of student aid on participation in higher education. Econ Educ Rev. 2014 Dec;43:66–78.

44. Gaughan J, Gutacker N, Grasic K, Kreif N, Siciliani L, Street A. Paying for efficiency: Incentivising same-day discharges in the English NHS. J Health Econ. 2019;68(8410622, jhe):102226.

45. Haghpanahan H, Lewsey J, Mackay DF, McIntosh E, Pell J, Jones A, et al. An evaluation of the effects of lowering blood alcohol concentration limits for drivers on the rates of road traffic accidents and alcohol consumption: a natural experiment. The Lancet. 2019;393(10169):321–9.

46. Mohan G, Longo A, Kee F. Evaluation of the health impact of an urban regeneration policy: Neighbourhood Renewal in Northern Ireland. J Epidemiol Community Health. 2017;(i1p, 7909766).

47. Serrano-Alarcon M, Kentikelenis A, McKee M, Stuckler D. Impact of COVID-19 lockdowns on mental health: Evidence from a quasi-natural experiment in England and Scotland. Health Econ. 31(2):284–96.

48. So V, Millard AD, Katikireddi SV, Forsyth R, Allstaff S, Deluca P, et al. Intended and unintended consequences of the implementation of minimum unit pricing of alcohol in Scotland: a natural experiment. 2021;

49. Szatkowski L, Coleman T, McNeill A, Lewis S. The impact of the introduction of smoke-free legislation on prescribing of stop-smoking medications in England. Addict Abingdon Engl. 2011;106(10):1827–34.

50. Ahlfeldt G, Roth D, Seidel T. The regional effects of Germany’s national minimum wage. Econ Lett. 2018;172:127–30.

51. Akbulut-Yuksel M, Khamis M, Yuksel M. Women make houses, women make homes. Labour Econ. 2017 Dec;49:145–61.

52. Anger S., Kvasnicka M., Siedler T. One last puff? Public smoking bans and smoking behavior. J Health Econ. 2011;30(3):591–601.

53. Kümpel C. Do financial incentives influence the hospitalization rate of nursing home residents? Evidence from Germany. Health Econ U K. 2019;28(11):1235–47.

54. Xu M, Bittschi B. Does the abolition of copayment increase ambulatory care utilization?: a quasi-experimental study in Germany. Eur J Health Econ. 2022 Nov;23(8):1319–28.

55. Chyderiotis S, Beck F, Andler R, Hitchman SC, Benmarhnia T. How to reduce biases coming from a before and after design: the impact of the 2007-08 French smoking ban policy. Eur J Public Health. 2019;29(2):372–7.

56. Ben Lakhdar C., Vaillant N.G., Wolff F.-C. Does smoke cross the border? Cigarette tax avoidance in France. Eur J Health Econ. 2016;17(9):1073–89.

57. Clark AE, Lepinteur A. A natural experiment on job insecurity and fertility in France. Rev Econ Stat. 2022 Mar;104(2):386–98.

58. Saffer H, Lamiraud K. The effect of hours of work on social interaction. Rev Econ Househ. 2012 Jun;10(2):237–58.

59. De Jorge-Huertas V, De Jorge-Moreno J. Analysis of the effects of (de)regulation on housing prices in Spain 1977-2019. J Econ Stud. 2021;48(6):1194–206.

60. García-Pérez JI, Marinescu I, Vall Castello J. Can fixed-term contracts put low skilled youth on a better career path? Evidence from Spain. Econ J. 2019;129(620):1693–730.

61. Maynou L., Coll-de-Tuero G., Saez M. The effects of copayment in primary health care: evidence from a natural experiment. Eur J Health Econ. 2019;20(8):1237–48.

62. Vall Castello J. Promoting Employment of Disabled Women in Spain: Evaluating a Policy. Labour Econ. 2012 Jan;19(1):82–91.

63. Bargain O, Gonzalez L, Keane C, Ozcan B. Female labor supply and divorce: new evidence from ireland. Eur Econ Rev. 2012 Nov;56(8):1675–91.

64. McDonnell T, Nicholson E, Bury G, Collins C, Conlon C, Denny K, et al. Policy of free GP care for children under 6 years: The impact on daytime and out-of-hours general practice. Soc Sci Med. 2022;296((McDonnell, Nicholson, Conlon, McAuliffe) IRIS Centre, School of Nursing, University College Dublin, Midwifery&Health Systems, Ireland):114792.

65. Stallings-Smith S, Zeka A, Goodman P, Kabir Z, Clancy L. Reductions in Cardiovascular, Cerebrovascular, and Respiratory Mortality following the National Irish Smoking Ban: Interrupted Time-Series Analysis. PLoS ONE. 2013;8(4):e62063.

66. Armeni P., Jommi C., Otto M. The simultaneous effects of pharmaceutical policies from payers’ and patients’ perspectives: Italy as a case study. Eur J Health Econ. 2016;17(8):963–77.

67. Fiorio CV, Siciliani L. Co-payments and the demand for pharmaceuticals: evidence from Italy. Econ Model. 2010 Jul;27(4):835–41.

68. Focacci CN. ‘You reap what you sow’: do active labour market policies always increase job security? Evidence from the youth guarantee. Eur J Law Econ. 2020 Jun;49(3):373–429.

69. Honkaniemi H, Katikireddi SV, Rostila M, Juarez SP. Psychiatric consequences of a father’s leave policy by nativity: a quasi-experimental study in Sweden. J Epidemiol Community Health. 2022;76(4):367–73.

70. Pettersson B., Hoffmann M., Andersson D., Wandell P., Levin L.-T. Utilization and costs of glucose lowering therapies following health technology assessment for the new reimbursement scheme in Sweden. Health Policy. 2012;108(2–3):207–15.

71. Runst P, Thonipara A. Dosis facit effectum why the size of the carbon tax matters: evidence from the Swedish residential sector. Energy Econ [Internet]. 2020 Sep;91. Available from: https://search.ebscohost.com/login.aspx?direct=true&db=ecn&AN=1861614&site=ehost-live

72. Daysal N.M., Trandafir M., van Ewijk R. Low-risk isn’t no-risk: Perinatal treatments and the health of low-income newborns. J Health Econ. 2019;64((Daysal, Trandafir) Department of Business and Economics, University of Southern Denmark, Campusvej 55, Odense M 5230, Denmark):55–67.

73. Hengel KO, Riumallo-Herl C, Schram J, Nieboer D, Van Der Beek A, Burdorf L. Effects of changes in early retirement policies on labor force participation: The differential effects for vulnerable groups. Occup Environ Med. 2021;78(SUPPL 1):A1.

74. Troelstra S.A., Bosdriesz J.R., De Boer M.R., Kunst A.E. Effect of tobacco control policies oninformation seeking for smoking cessation in the Netherlands: A google trends study. PLoS ONE. 2016;11(2):e0148489.

75. Zoidze A., Rukhazde N., Chkhatarashvili K., Gotsadze G. Promoting universal financial protection: health insurance for the poor in Georgia--a case study. Health Res Policy Syst BioMed Cent. 2013;11((Zoidze) Curatio International Foundation, P,O, Box 110, Tbilisi 380079, Georgia.).

76. Nedberg IH, Manjavidze T, Rylander C, Blix E, Skjeldestad FE, Anda EE. Changes in cesarean section rates after introduction of a punitive financial policy in Georgia: A population-based registry study 2017-2019. PLoS ONE. 2022;17(7 July):e0271491.

77. Gambaryan M, Reeves A, Deev A, Popovich M, Drapkina O, Snell A, et al. Effects of tobacco control policy on cardiovascular morbidity and mortality in Russia. Eur J Public Health. 2018;28(2 Supplement):14–6.

78. Shelkova NY. Stronger women, better men? Family bargaining and public policy in contemporary Russia. Rev Econ Househ. 2020 Jun;18(2):335–55.

79. Boes S., Marti J., MacLean J.C. The impact of smoking bans on smoking and consumer behavior: quasi-experimental evidence from switzerland. Health Econ U K. 2015;24(11):1502–16.

80. Dumeignil C. The impact of cross-border labor mobility on real estate price trends: a natural experiment. Int Reg Sci Rev. 2022 Jan;45(1):108–32.

81. Wicki M, Gmel G. Hospital admission rates for alcoholic intoxication after policy changes in the canton of Geneva, Switzerland. Drug Alcohol Depend. 2011;118(2–3):209–15.

82. Temkin E., Schwaber M.J., Vaturi A., Nadir E., Zilber R., Barel O., et al. Effect of a national policy of universal masking and uniform criteria for severe acute respiratory coronavirus virus 2 (SARS-CoV-2) exposure on hospital staff infection and quarantine. Infect Control Hosp Epidemiol. 2022;43(6):757–63.

83. Bratberg E., Holmas T.H., Monstad K. Health effects of reduced workload for older employees. Health Econ U K. 2020;29(5):554–66.

84. Muravyev A, Talavera O. Can state language policies distort students’ demand for education? J Comp Econ. 2016 May;44(2):383–99.

85. Grenet J. Is extending compulsory schooling alone enough to raise earnings? Evidence from French and British compulsory schooling laws. Scand J Econ. 2013 Jan;115(1):176–210.

86. Reinhold S, Kneip T, Bauer G. The long run consequences of unilateral divorce laws on children; evidence from SHARELIFE. J Popul Econ. 2013 Jul;26(3):1035–56.

87. Lavikainen P, Aarnio EJ, Jalkanen K, Tirkkonen H, Rautiainen P, Laatikainen T, et al. Impact of co-payment level increase of antidiabetic medications on glycaemic control: an interrupted time-series study among Finnish patients with type 2 diabetes. Value Health. 2020;22:S586–7.

88. Biro A. Reduced user fees for antibiotics under age 5 in Hungary: Effect on antibiotic use and imbalances in the implementation. PLoS ONE. 2019;14(6):e0219085.

89. Kleif HB, Nielsen Arendt J. Incentive effects of cash benefit among low-skilled young adults: Applying a regression discontinuity design. PloS One. 2020;15(11):e0241279.

90. Kaliskova K. Labor supply consequences of family taxation: evidence from the Czech Republic. Labour Econ. 2014 Oct;30:234–44.

91. Grabovac I, Hochfellner L, Rieger M, Jewell J, Snell A, Weber A, et al. Impact of Austria’s 2009 trans fatty acids regulation on all-cause, cardiovascular and coronary heart disease mortality. Eur J Public Health. 2018;28(2 Supplement):4–9.

92. Hamilton I, Lloyd C, Hewitt C, Godfrey C. Effect of reclassification of cannabis on hospital admissions for cannabis psychosis: A time series analysis. Int J Drug Policy. 2014;25(1):151–6.

93. Madani MT, Madani L, Ghogomu ET, Dahrouge S, Hébert PC, Juando-Prats C, et al. Is equity considered in systematic reviews of interventions for mitigating social isolation and loneliness in older adults? BMC Public Health. 2022 Dec 1;22(1):2241.

94. Jull J, Whitehead M, Petticrew M, Kristjansson E, Gough D, Petkovic J, et al. When is a randomised controlled trial health equity relevant? Development and validation of a conceptual framework. BMJ Open. 2017 Sep 1;7(9):e015815.

95. Miani C, Wandschneider L, Niemann J, Batram-Zantvoort S, Razum O. Measurement of gender as a social determinant of health in epidemiology—A scoping review. PLoS ONE. 2021 Nov 3;16(11):e0259223.

96. Petticrew M, Tugwell P, Kristjansson E, Oliver S, Ueffing E, Welch V. Damned if you do, damned if you don’t: subgroup analysis and equity. J Epidemiol Community Health. 2012 Jan 1;66(1):95–8.

97. Backholer K, Beauchamp A, Ball K, Turrell G, Martin J, Woods J, et al. A Framework for Evaluating the Impact of Obesity Prevention Strategies on Socioeconomic Inequalities in Weight. Am J Public Health. 2014 Oct;104(10):e43–50.

98. Kelly-Irving M, Ball WP, Bambra C, Delpierre C, Dundas R, Lynch J, et al. Falling down the rabbit hole? Methodological, conceptual and policy issues in current health inequalities research. Crit Public Health. 2023 Jan 1;33(1):37–47.
