# Supplementary figures and images for: How is health equity considered in policy evaluations employing quasi-experimental methods? A scoping review and content analysis
Source: Eur J Public Health. 2024 Nov 27;35(1):42–51. doi: 10.1093/eurpub/ckae188 (PMC11832135; doi:10.1093/eurpub/ckae188)

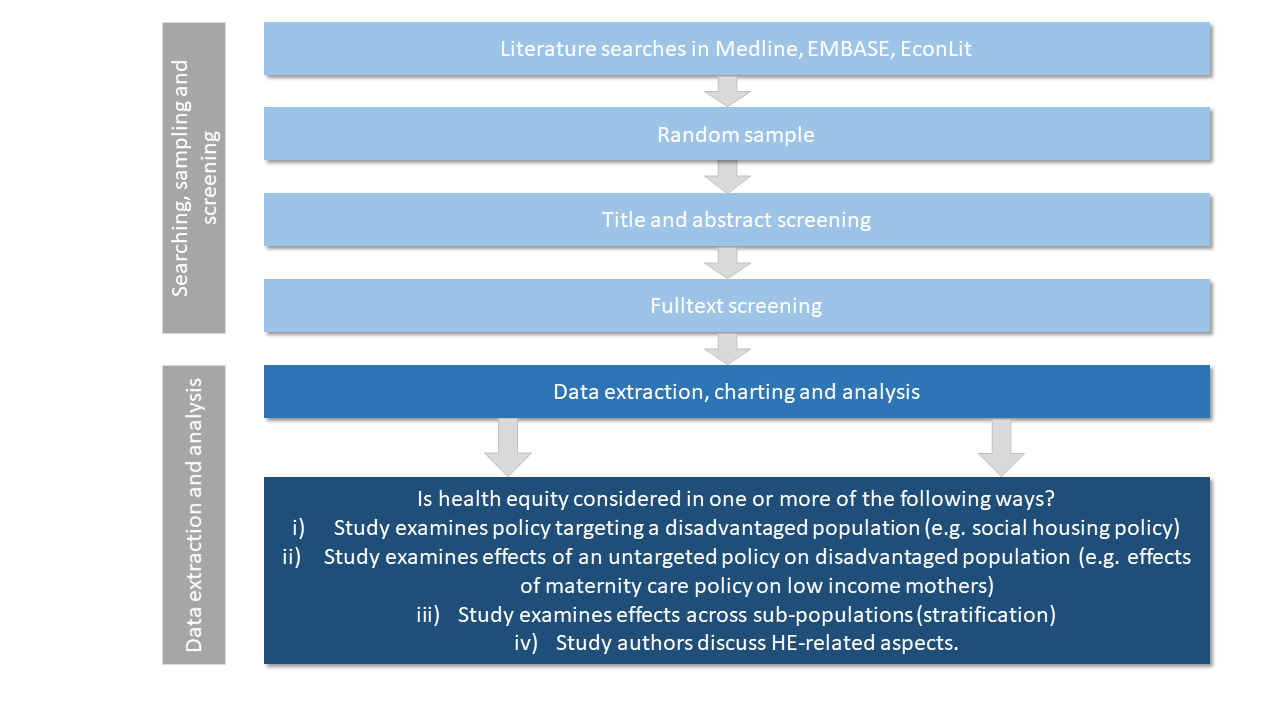

Supplement: ckae188_Supplementary_Data [file ckae188_supplementary_data.zip › ckae188_Supplementary_Data/ejph-2024-08-om-0528-File006.tif]
